# Toxicity and Bioremediation of Heavy Metals Contaminated Ecosystem from Tannery Wastewater: A Review

**DOI:** 10.1155/2018/2568038

**Published:** 2018-09-27

**Authors:** Bernard E. Igiri, Stanley I. R. Okoduwa, Grace O. Idoko, Ebere P. Akabuogu, Abraham O. Adeyi, Ibe K. Ejiogu

**Affiliations:** ^1^Chemical and Biochemical Remediation Unit, Directorate of Research and Development, Nigerian Institute of Leather and Science Technology, Zaria 810001, Kaduna State, Nigeria; ^2^Infohealth Awareness Department, SIRONigeria Global Limited, Abuja 900001, FCT, Nigeria

## Abstract

The discharge of untreated tannery wastewater containing biotoxic substances of heavy metals in the ecosystem is one of the most important environmental and health challenges in our society. Hence, there is a growing need for the development of novel, efficient, eco-friendly, and cost-effective approach for the remediation of inorganic metals (Cr, Hg, Cd, and Pb) released into the environment and to safeguard the ecosystem. In this regard, recent advances in microbes-base heavy metal have propelled bioremediation as a prospective alternative to conventional techniques. Heavy metals are nonbiodegradable and could be toxic to microbes. Several microorganisms have evolved to develop detoxification mechanisms to counter the toxic effects of these inorganic metals. This present review offers a critical evaluation of bioremediation capacity of microorganisms, especially in the context of environmental protection. Furthermore, this article discussed the biosorption capacity with respect to the use of bacteria, fungi, biofilm, algae, genetically engineered microbes, and immobilized microbial cell for the removal of heavy metals. The use of biofilm has showed synergetic effects with many fold increase in the removal of heavy metals as sustainable environmental technology in the near future.

## 1. Introduction

Industrial tannery wastewater is a major source of heavy metal contamination in our environment. Heavy metals are of economic significance in industrial use and the most important pollutants in the environment. Environmental pollution by heavy metals has become a serious threat to living organisms in an ecosystem [1–5]. Metal toxicity is of great environmental concern because of their bioaccumulation and nonbiodegradability in nature [6, 7]. Several inorganic metals like magnesium (Mg), nickel (Ni), chromium (Cr^3+^), copper (Cu), calcium (Ca), manganese (Mn), and sodium (Na) as well as zinc (Zn) are vital elements needed in small quantity for metabolic and redox functions. Heavy metals such as aluminium (Al), lead (Pb), cadmium (Cd), gold (Au), mercury (Hg), and silver (Ag) do not have any biological role and are toxic to living organisms [1, 8, 9].

Bioremediation is employed in order to transform toxic heavy metals into a less harmful state using microbes [10–12] or its enzymes to clean-up polluted environment [13]. The technique is environmentally friendly and cost-effective in the revitalization of the environment [3, 9, 14]. Bioremediation of heavy metals has limitations. Among these are production of toxic metabolites by microbes and nonbiodegradability of heavy metals.

The direct use of microorganisms with distinctive features of catabolic potential and/or their products such as enzymes and bio surfactant is a novel approach to enhance and boost their remediation efficacy [15, 16]. Different alternatives have also been anticipated to widen the applications of microbiological techniques towards the remediation of heavy metals. For instance, the use of microbial fuel cell (MFC) to degrade recalcitrant heavy metals has been explored. Biofilm-mediated bioremediation can be applied for cleaning up of heavy metal contaminated environment.

Microbial technologies are active and growing [17]. Long trajectory exists on how microbes and metals interact in both natural and man-made environments. Microbial-metal interactions is primarily focused on metals removal, i.e., remediation and depollution. The recent revival of the use of solid-state electrodes as electron donors or acceptors for microbial growth has brought innovative prospects, resulting to microbial-electrochemical technologies (METs) [18]. The application of microorganisms as a green approach for the synthesis of metallic nanoparticles (NPs) has been reported [19]. Genetically modified microorganisms have also been used as a remediation technique [20, 21]. Genetic engineering and chemical modification could alter the components of cells surface and can efficiently improve the adsorption capacity and selectivity to target-metal species.

Several factors which influences and limit bioremediation efficiency include temperature, pH, redox potential, nutritional status, moisture, and chemical composition of heavy metals [22]. The use of microbes alone has shown limited efficiency owing to various factors including poor competitiveness as well as excessive heavy metal concentrations. Effectiveness can be enhanced by several amendments with inorganic nutrients, biosurfactants, bulking agents, and compost as well as biochar [23]. These adjustments have been comprehensively reviewed in recent studies [24–26].

There are several protection mechanisms of heavy metal resistance by microbial cells. These mechanisms are extracellular barrier, extracellular sequestration, and active transport of metal ions (efflux), intracellular sequestration, and reduction of metal ions [27, 28].

This study therefore seeks to review the reports of previous investigators on the toxic effect and the use of microbial cell and their products, namely, biosurfactants, to enhance remediation of heavy metals. It also discusses the factors that influence bioremediation of heavy metals along with their underlining mechanisms. The findings and analyses are presented in the following sections. Current research work on microbial biosorption and detoxification is not only summarized but also future directions are suggested.

## 2. Research Methodology

### 2.1. Search Strategy

Relevant scientific literatures from major databases were searched for original research articles on the toxic effects of heavy metals and the use of microbial cell to remediate heavy metals. The following databases were searched: PubMed, ScienceDirect, and Google Scholar. The keyword combinations for the search were toxicity of heavy metals, tannery effluent, and biofilms, factors that affect microbial remediation, bioremediation, and mechanisms of microbial remediation.

### 2.2. Inclusion Criteria

Original scientific research studies that reported on the toxic effects of heavy metals and the use of microorganisms to clean up heavy metal in the ecosystem were included.

### 2.3. Exclusion Criteria

Articles that reported on the bioremediation of organic compounds, phytoremediation of heavy metals, and other biological techniques were excluded.

## 3. Toxicity of Heavy Metals to Microorganisms

Toxicity of heavy metals is the ability of a metal to cause detrimental effects on microorganisms, and it depends on the bioavailability of heavy metal and the absorbed dose [29]. Heavy metal toxicity involves several mechanisms, that is, breaking fatal enzymatic functions, reacting as redox catalysts in the production of reactive oxygen species (ROS), destructing ion regulation, and directly affecting the formation of DNA as well as protein [30, 31]. The physiological and biochemical properties of microorganisms can be altered by the presence of heavy metals. Chromium (Cr) and cadmium (Cd) are capable of inducing oxidative damage and denaturation of microorganisms as well as weakening the bioremediation capacity of microbes.

Chromium Cr (III) may change the structure and activity of enzymes by reacting with their carboxyl and thiol groups [32]. Intracellular cationic Cr (III) complexes interact electrostatically with negatively charged phosphate groups of DNA, which could affect transcription, replication, and cause mutagenesis [32].

Heavy metals like copper (Cu (I) and Cu (II)) could catalyse the production of ROS via Fenton and Haber-Weis reactions, which will act as soluble electron carries. This can cause severe injury to cytoplasmic molecules, DNA, lipids, and other proteins [33, 34]. Aluminium (Al) could stabilize superoxide radicals, which is responsible for DNA damage [35]. Heavy metals could stop vital enzymatic functions by competitive or noncompetitive interactions with substrates that will cause configurational changes in enzymes [30]. Furthermore, it can also cause ion imbalance by adhering to the cell surface and entering through ion channels or transmembrane carriers [36].

Cadmium (Cd) and lead (Pb) pose deleterious effect on microbes, damage cell membranes, and destroy the structure of DNA. This harmfulness is generated by the displacement of metals from their native binding sites or ligand interactions [37]. The morphology, metabolism, and growth of microbes are affected by changing the nucleic acid structure, causing functional disturbance, disrupting cell membranes, inhibiting enzyme activity, and oxidative phosphorylation [38, 39] (Table 1).

## 4. Factors Affecting Microbial Remediation of Heavy Metals

The propensity of heavy metals to be stimulatory or inhibitory to microorganisms is determined by the total metal ion concentrations, chemical forms of the metals, and related factors such as redox potential. Environmental factors like temperature, pH, low molecular weight organic acids, and humic acids can alter the transformation, transportation, valance state of heavy metals, and the bioavailability of heavy metals towards microorganisms. Heavy metals tend to form free ionic species at acidic pH levels, with more protons available to saturate metal-binding sites. At higher hydrogen ion concentrations, the adsorbent surface is more positively charged, hence reducing the attraction between adsorbent and metal cations thereby increasing its toxicity.

Temperature plays a significant role in the adsorption of heavy metals. Increase in temperature increases the rate of adsorbate diffusion across the external boundary layer. The solubility of heavy metals increases with an increase in temperature, which improves the bioavailability of heavy metals [44]. However, the actions of microorganisms increase with rise in temperature at a suitable range, and it enhances microbial metabolism and enzyme activity, which will accelerate bioremediation. The stability of microbes-metal complex depends on the sorption sites, microbial cell wall configuration, and ionization of chemical moieties on the cell wall. The outcome of degradation process depends on the substrate and range of environmental factors (Table 2).

## 5. Mechanism of Microbial Detoxification of Heavy Metal

Microorganisms adopt different mechanisms to interact and survive in the presence of inorganic metals. Various mechanisms used by microbes to survive metal toxicity are biotransformation, extrusion, use of enzymes, production of exopolysaccharide (EPS) [41, 46], and synthesis of metallothioneins. In response to metals in the environment, microorganisms have developed ingenious mechanisms of metal resistance and detoxification. The mechanism involves several procedures, together with electrostatic interaction, ion exchange, precipitation, redox process, and surface complexation [47]. The major mechanical means to resist heavy metals by microorganism are metal oxidation, methylation, enzymatic decrease, metal-organic complexion, metal decrease, metal ligand degradation, metal efflux pumps, demethylation, intracellular and extracellular metal sequestration, exclusion by permeability barrier, and production of metal chelators like metallothioneins and bio surfactants [48].

Microorganisms can decontaminate metals by valence conversion, volatilization, or extracellular chemical precipitation [48]. Microorganisms have negative charge on their cell surface because of the presence of anionic structures that empower the microbes to bind to metal cations [49]. The negatively charged sites of microbes involved in adsorption of metal are the hydroxyl, alcohol, phosphoryl, amine, carboxyl, ester, sulfhydryl, sulfonate, thioether, and thiol groups [49].

### 5.1. Bio Sorption Mechanism

**Figure 1:**
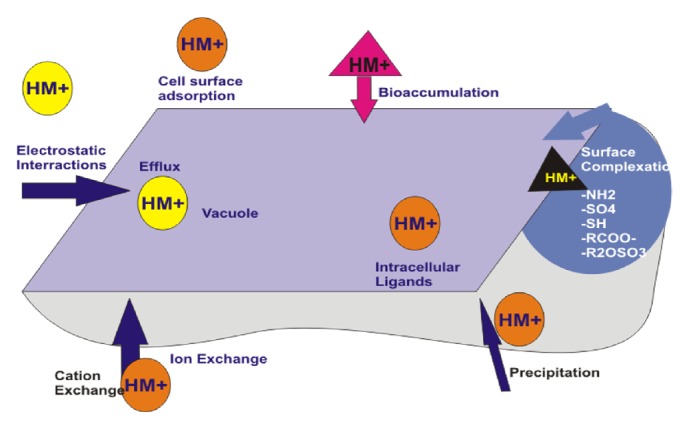
Mechanisms of heavy metal uptake by microorganisms [47, 53].

The uptake of heavy metals by microbial cells through biosorption mechanisms can be classified into metabolism-independent biosorption, which mostly occurs on the cells exterior and metabolism-dependent bioaccumulation, which comprises sequestration, redox reaction, and species-transformation methods [50, 51]. Bio sorption can be carried out by dead biomass or living cells as passive uptake through surface complexation onto the cell wall and surface layers [52]. Bioaccumulation depends on a variety of chemical, physical, and biological mechanisms (Figure 1) and these factors are intracellular and extracellular processes, where biosorption plays a limited and ill-defined role [52].

### 5.2. Intracellular Sequestration

Intracellular sequestration is the complexation of metal ions by various compounds in the cell cytoplasm. The concentration of metals within microbial cells can result from interaction with surface ligands followed by slow transport into the cell. The ability of bacterial cells to accumulate metals intracellular has been exploited in practices, predominantly in the treatment of effluent treatment. Cadmium-tolerant* P. putida *strain possessed the ability of intracellular sequestration of copper, cadmium, and zinc ions with the help of cysteine-rich low molecular weight proteins [54]. Also, intracellular sequestration of cadmium ions by glutathione was revealed in* Rhizobium leguminosarum *cells [55].

The rigid cell wall of fungi is made up of chitin, mineral ions, lipids, nitrogen-containing polysaccharide, polyphosphates, and proteins. They can decontaminate metal ions by energetic uptake, extracellular and intracellular precipitation, and valence conversion, with several fungi accumulating metals to their mycelium and spores. The exterior of the cell wall of fungi behaves like a ligand used for labelling metal ions and brings about the elimination of inorganic metals [56–59]. Peptidoglycan, polysaccharide, and lipid are components of cell wall that are rich in metal-binding ligands (e.g., -OH, -COOH, -HPO42−, SO42− -RCOO−, R2OSO3−, -NH2, and -SH). Amine can be more active in metal uptake among these functional groups, as it binds to anionic metal species via electrostatic interaction and cationic metal species through surface complexation.

### 5.3. Extracellular Sequestration

Extracellular sequestration is the accumulation of metal ions by cellular components in the periplasm or complexation of metal ions as insoluble compounds. Copper-resistant* Pseudomonas syringae *strains produced copper-inducible proteins CopA, CopB (periplasmic proteins), and CopC (outer membrane protein) which bind copper ions and microbial colonies [60]. Bacteria can eject metal ions from the cytoplasm to sequester the metal within the periplasm. Zinc ions can cross from the cytoplasm by efflux system where they are accumulated in the periplasm of Synechocystis PCC 6803 strain [61].

Metal precipitation is an extracellular sequestration. Iron reducing bacterium such as* Geobacter* spp. and sulfur reducing bacterium like* Desulfuromonas *spp. are capable of reducing harmful metals to less or nontoxic metals.* G. metallireducens*, a strict anaerobe, is capable of reducing manganese (Mn), from lethal Mn (IV) to Mn (II), and uranium (U), from poisonous U (VI) to U (IV) [49].* G. sulfurreducens* and* G. metallireducens *have the ability to decrease chromium (Cr) from the very lethal Cr (VI) to less toxic Cr (III) [62]. Sulfate-reducing bacteria generate large amounts of hydrogen sulfide that causes precipitation of metal cations [63, 64].

*Klebsiella planticola *strain generates hydrogen sulfide from thiosulfate under anaerobic conditions and precipitated cadmium ions as insoluble sulfides [65]. Also, cadmium was precipitated by* P. aeruginosa *strain under aerobic conditions [66].* Vibrio harveyi *strain precipitated soluble divalent lead as complex lead phosphate salt [67].

### 5.4. Extracellular Barrier of Preventing Metal Entry into Microbial Cell

Microbial plasma membrane, cell wall, or capsule could prevent metal ions from entering the cell. Bacteria can adsorb metal ions by ionizable groups of the cell wall (amino, carboxyl, phosphate, and hydroxyl groups) [68, 69]. Pardo* et al.* [70], Taniguchi* et al.* [69], and Green-Ruiz [71] observed high level of passive biosorption of heavy metal ions for nonviable cells of* Pseudomonas putida, Brevibacterium *sp., and* Bacillus *sp.

*Pseudomonas aeruginosa *biofilm cells show higher resistance to ions of copper, lead, and zinc than planktonic cells, while cells located at the periphery of the biofilm were killed. Extracellular polymers of biofilm accumulated metal ions and then protect bacterial cells inside the biofilm [72].

### 5.5. Methylation of Metals

Methylation increases metal toxicity as a result of increased lipophilicity and thus increased permeation across cell membranes. Microbial methylation plays a significant function in metal remediation. Methylated compounds are regularly explosive; for instance, Hg (II) can be bio methylated by some bacteria such as* Bacillus *spp*., Escherichia *spp.*, Clostridium *spp.,* and Pseudomonas *spp. to gaseous methyl mercury. Bio methylation of selenium (Se) to volatile dimethyl selenide and arsenic (As) to gaseous arsines as well as lead (Pb) to dimethyl lead was witnessed in polluted top soil [48].

### 5.6. Reduction of Heavy Metal Ions by Microbial Cell

Microbial cells can convert metal ion from one oxidation state to another, hence reducing their harmfulness [73]. Bacteria use metals and metalloids as electron donors or acceptors for energy generation. Metals in the oxidized form could serve as terminal acceptors of electrons during anaerobic respiration of bacteria. Reduction of metal ions through enzymatic activity could result in formation of less toxic form of mercury and chromium [74, 75].

## 6. Bioremediation Capacity of Microorganisms on Heavy Metals

The uptake of heavy metals by microorganisms occurs via bioaccumulation which is an active process and/or through adsorption, which is a passive process. Several microorganisms like bacteria, fungi, and algae have been used to clean up heavy metal contaminated environments (Table 3) [76, 77]. The application of metal-resistant strains in single, consortium, and immobilized form for the remediation of heavy metals has yielded effective results while the immobilized form could have more chemosorption sites to biosorb heavy metals.

### 6.1. Bacteria Remediation Capacity of Heavy Metal

Microbial biomass has different biosorptive abilities, which also varies significantly among microbes. However, the biosorption ability of each microbial cell depends on its pretreatment and the experimental conditions. Microbial cell must adapt to alteration of physical, chemical and bioreactor configuration to enhance biosorption [52]. Bacteria are important biosorbents due to their ubiquity, size, and ability to grow under controlled conditions and resilience to environmental conditions [78, 79].

De Jaysankar and his coauthors [99] use mercury-resistant bacteria such as* Alcaligenes faecalis, Bacillus pumilus, Pseudomonas aeruginosa, *and* Brevibacterium iodinium *for the removal of cadmium (Cd) and lead (Pb). In this study,* P. aeruginosa *and* A. faecalis* removed 70 % and 75 % cadmium (Cd) with reduction of 1000 mg/L to 17.4 mg/L of cadmium (Cd) by* P. aeruginosa *and to 19.2 mg/L by* A. faecalis* in about 72hrs.* Brevibacterium iodinium *and* Bacillus pumilus* remove greater than 87 % and 88 % of lead (Pb) with a reduction of 1000 mg/L to 1.8 mg/L in 96 hours (Table 3). In another study, [118] uses indigenous facultative anaerobic* Bacillus cereus* to detoxify hexavalent chromium.* Bacillus cereus *has an excellent capacity of 72 % Cr (VI) removal at 1000 *μ*g/mL chromate concentration. The bacteria were capable of reducing Cr (VI) under a wide range of temperatures (25 to 40°C) and pH (6 to 10) with optimum at 37°C and initial pH 8.0.

Several heavy metals have been tested using bacteria species like* Flavobacterium*,* Pseudomonas*,* Enterobacter, Bacillus,* and* Micrococcus* sp. (Table 3). Their great biosorption ability is due to high surface-to-volume ratios and the potential active chemosorption sites (teichoic acid) on the cell wall [119]. Bacteria are more stable and survive better when they are in mixed culture [120]. Therefore, consortia of cultures are metabolically superior for biosorption of metals and are more appropriate for field application [121]. De Jaysankar* et al.* [99] reported 78 % reduction of chromium (Cr) using bacteria consortium of* Acinetobacter* sp. and* Arthrobacter* sp. of 16 mg/L metal ion concentration.* Micrococcus luteus* was used to remove a huge quantity of Pb from a synthetic medium. Under ideal environments, the elimination ability was 1965 mg/g [122].

Abioye and his coworkers [123] investigated the biosorption of lead (Pb), chromium (Cr), and cadmium (Cd) in tannery effluent using* Bacillus subtilis, B. megaterium, Aspergillus niger, and Penicillium *sp.* B. megaterium* recorded the highest lead (Pb) reduction (2.13 to 0.03 mg/L), followed by* B*.* subtilis* (2.13-0.04 mg/L).* A. niger* show the highest ability to reduce the concentration of chromium (Cr) (1.38-0.08 mg/L) followed by* Penicillium* sp. (1.38-0.13 mg/L) while* B. subtilis* exhibited the highest ability to reduce the concentration of cadmium (Cd) (0.4-0.03 mg/L) followed by* B. megaterium* (0.04-0.06 mg/L) after 20 days. Kim and his coauthors [76], designed a batch system using zeolite-immobilized* Desulfovibrio desulfuricans* for the removal of chromium (Cr^6+^), copper (Cu), and nickel (Ni) with removal efficiency of 99.8%, 98.2%, and 90.1%, respectively ( Table 3). Ashruta and his coworkers [124] reported efficient removal of chromium, zinc, cadmium, lead, copper, and cobalt by bacterial consortia at approximately 75 to 85% in less than two hours of contact duration.

### 6.2. Fungi Remediation Capacity of Heavy Metal

Fungi are widely used as biosorbents for the removal of toxic metals with excellent capacities for metal uptake and recovery [125–127]. Most studies showed that active and lifeless fungal cells play a significant role in the adhesion of inorganic chemicals [111, 128–130]. Srivastava and Thakur [131] also reported the efficiency of* Aspergillus* sp. used for the removal of chromium in tannery waste water. 85% of chromium was removed at pH 6 in a bioreactor system from the synthetic medium, compared to a 65 % removal from the tannery effluent. This could be due to the presence of organic pollutants that hinder the growth of the organism.

*Coprinopsis atramentaria* is studied for its ability to bioaccumulate 76 % of Cd^2+^, at a concentration of 1 mg L−1 of Cd^2+^, and 94.7% of Pb^2+^, at a concentration of 800 mg L−1 of Pb^2+^. Therefore, it has been documented as an effective accumulator of heavy metal ions for mycoremediation [132]. Park and his coauthors [133] reported that dead fungal biomass of* Aspergillus niger, Rhizopus oryzae, Saccharomyces cerevisiae*, and* Penicillium chrysogenum* could be used to convert toxic Cr (VI) to less toxic or nontoxic Cr (III). Luna* et al.* [134] also observed that* Candida sphaerica* produces biosurfactants with a removal efficiency of 95 %, 90 %, and 79 % for Fe (iron), zinc (Zn), and lead (Pb), respectively. These surfactants could form complexes with metal ions and interact directly with heavy metals before detachment from the soil.* Candida* spp. accumulate substantial quantity of nickel Ni (57–71%) and copper Cu (52– 68 %), but the process was affected by initial metal ion concentration and pH (optimum 3–5) [135].

Biosurfactants have gained interest in recent years owing to their low toxicity, biodegradable nature, and diversity. Mulligan* et al*. [136] assessed the viability of using surfactin, rhamnolipid, and sophorolipid for the removal of heavy metals (Cu and Zn). A single washing with 0.5 % rhamnolipid removed 65 % of copper (Cu) and 18 % of the zinc (Zn), whereas 4% sophorolipid removed 25% of the copper (Cu) and 60% of zinc (Zn). Several strains of yeast such as* Hansenula polymorpha*,* S. cerevisiae, Yarrowia lipolytica, Rhodotorula pilimanae, Pichia guilliermondii*, and* Rhodotorula mucilage* have been used to bio-convert Cr (VI) to Cr (III) [137–139].

### 6.3. Heavy Metal Removal Using Biofilm

There are several reports on the application of biofilms for the removal of heavy metals. Biofilm acts as a proficient bioremediation tool as well as biological stabilization agent. Biofilms have very high tolerance against toxic inorganic elements even at a concentration that is lethal. It was revealed in a study conducted on* Rhodotorula mucilaginosa* that metal removal efficiency was from 4.79 to 10.25 % for planktonic cells and from 91.71 to 95.39 % for biofilm cells [140]. Biofilms mechanisms of bioremediation could either be via biosorbent or by exopolymeric substances present in biofilms which contain molecules with surfactant or emulsifier properties [141].

### 6.4. Algae Remediation Capacity of Heavy Metal

Algae are autotrophic and hence require low nutrients and produce enormous biomass compared to other microbial biosorbents. These biosorbents have also been used for heavy metal removal with a high sorption capacity [12]. Algae biomass is used for bioremediation of heavy metal polluted effluent via adsorption or by integration into the cells. Phycoremediation is the use of various types of algae and cyanobacteria for the remediation of heavy metals by either removal or degradation of toxicant [142]. Algae have various chemical moieties on their surface such as hydroxyl, carboxyl, phosphate, and amide, which act as metal-binding sites [12, 143].

Goher and his coauthors [113] used dead cells of* Chlorella vulgaris* to remove cadmium (Cd^2+^), copper (Cu^2+^), and lead (Pb^2+^) ions from aqueous solution under various conditions of pH, biosorbent dosage, and contact time. The results suggested that the biomass of* C. vulgaris* is an extremely efficient biosorbent for the removal of cadmium (Cd^2+^)^,^ copper (Cu^2+^) and lead (Pb^2+^) at 95.5 %, 97.7 %, and 99.4 %, respectively, from mixed solution of 50 mg dm^−3^ of each metal ion (Table 3).

### 6.5. Immobilized Biosorption of Heavy Metal

The use of encapsulated biomass enhances biosorption performance and increases its physical and chemical stability. Immobilizations of microbial biomass in polymeric matrixes confer rigidity and heat resistivity with optimum porosity for practical applications.* Agrobacterium *biomass was encapsulated in alginate with iron oxide nanoparticles and showed an adsorption capacity of 197.02 mg/g for Pb and was effective for five consecutive cycles [144].

### 6.6. Microbial Genetic Engineering

With the advanced in genetic engineering, microbes are engineered with desired characteristics such as ability to tolerate metal stress, overexpression of metal-chelating proteins and peptides, and ability of metal accumulation. Frederick* et al.* [145] engineered microorganisms to produce trehalose and establish that it reduces 1 mM Cr (VI) to Cr (III). Engineered* Chlamydomonas reinhardtii *generated significant increase in tolerance to Cd toxicity and its accumulation [146]. Genetically engineered microbes for heavy metal remediation involve the use of* Escherichia coli *(*E. coli* ArsR (ELP153AR)) to target As(III) [147] and* Saccharomyces cerevisiae* (CP2 HP3) to target Cd^2+^ and Zn^2+^ [148].* Corynebacterium glutamicum* was genetically modified using overexpression of* ars *operons (*ars*1 and* ars*2) to decontaminate As-contaminated sites [149].

Bioremediation of heavy metals has been extensively studied and the performance of several bioremediators were reviewed and summarized. Bioremediation is an environmentally friendly and cost-effective technology for the clean-up of complex industrial tannery effluent containing heavy metals. Many natural biosorbents of microbial origins have been identified with efficient biosorption characteristics. Recent surface modifications on these bioremediators have helped to ameliorate their metal-binding properties and increase the overall cost of the process. In spite of such short comings, both native and modified biosorbents have demonstrated their compatibility when tested with tannery effluent. These biosorbents showed effective metal removal over a wide range of temperature, pH, and solution conditions.

## 7. Future Outlook

Certain factors inhibiting the widespread application of this technology as identified by various researchers include difficulty in obtaining a reliable and inexpensive biomass and negative effects of coexisting metal ions on biosorptive capacity among others. Tannery effluent and biosorbent characteristics need to be assessed prior to application. Keeping in focus the inhibitions of bioremediation technology, the future prospect looks promising on microbial genetic technologies and the development of increased specificity using biofilms which could be achieved by optimization process and immobilization techniques. Hence, more effort should be made in biofilms mediated bioremediation, genetically modified microbes, and microbial fuel cell (MFC) in the bioremediation of heavy metals in the ecosystem.

## 8. Conclusion

The current states of the bioremediation of heavy metal reviewed in this study show much promise for metal biosorption and detoxification, especially from biofilm and genetically modified microbes. Biofilm-mediated techniques, microbial gene transfer, and microbial fuel cells-based techniques have come up as strong contenders in recent years. The peptidoglycan and polysaccharides component of the cell wall of the biosorbents is an active binding site for higher metal uptake. This technique is cost-effective and a green technology that has advantages such as faster kinetics, high metal binding over a broad range of pH, and temperature. This review provides an opportunity to reveal the role of microbial cell, biofilm, and their metabolites towards remediation of heavy metals and environmental research. Further research area needs to be extended on the focus of gene transfer within biofilms for heavy metal remediation. These would facilitate the development of improved techniques for the bioremediation of heavy metals in the ecosystem.

## Figures and Tables

**Table 1:** Toxicity of heavy metals to microorganisms.

**Heavy Metals **	**Effects on Microbes**	**Citations**
Arsenic	Deactivation of enzymes	[40]
Cadmium	Denature protein, destroy nucleic acid, hinder cell division and transcription	[38]
Chromium	Growth inhibition, elongation of lag phase, inhibition of oxygen uptake	[32]
Copper	Disrupt cellular function, inhibit enzyme activities	[38]
Selenium	Inhibits growth rate	[41]
Lead	Destroyed nucleic acid and protein, inhibit enzyme actions and transcription	[38]
Mercury	Denature protein, inhibit enzyme function, disrupt cell membrane	[38]
Nickel	Upset cell membrane, hinder enzyme activities and oxidative stress	[38, 42]
Silver	Cell lysis, inhibit cell transduction and growth	[43]
Zinc	Death, decrease in biomass, inhibits growth	[42]

**Table 2:** Factors that influence bioremediation of heavy metals [45].

Factors	Activities
Microbial	(i) Production of toxic metabolites
	(ii) Enzymes induction
	(iii) Mutation and horizontal gene transfer
	(iv) Enrichment of capable microbial populations

Substrate	(i) Chemical structure of contaminants
	(ii) Too low concentration of contaminants
	(iii) Toxicity of contaminants
	(iv) Solubility of contaminants

Environmental	(i) Inhibitory Environmental conditions
	(ii) Depletion of preferential substrates
	(iii) Lack of nutrients

Mass transfer limitations	(i) Oxygen diffusion and solubility
	(ii) Solubility/miscibility in/with water
	(iii) Diffusion of nutrients

Growth substrate vs. co-metabolism	(i) Microbial interaction( competition, succession, and predation)
	(ii) Concentration
	(iii) Alternate carbon source present

Biological aerobic vs. anaerobic process	(i) Microbial population present in the site
	(ii) Oxidation/reduction potential
	(iii) Availability of electron acceptors

**Table 3:** Remediation of heavy metal by microorganisms.

Microbial Group	Bioremediator	Metals	Metal ion Concentration (mg/L)	Sorption Efficiency (%)	Reference
Bacteria	*Acinetobacter *sp.	Cr	16	87	[80]
	*Sporosarcina saromensis* (M52)		50	82.5	[81]
	*Bacillus cereus*		1500	81	[82]
	*Bacillus cereus* (immobilized)		1500	96	
	*Bacillus circulans *MN1		1110	71.4	[83]
	*Bacillus cereus* plus 0.5 glucose		1	78	[77]
	*Bacillus cereus*		1	72	
	*Bacillus *sp. SFC		25	80	[84]
			50	43	
	*Bacillus subtilis*		0.57	99.6	[76]
	*Desulfovibrio desulfuricans *(KCTC 5768) (immobilize on zeolite)		100	99.8	[76]
			200	56.1	
			50	99.6	
	*Staphylococcus* sp.		4.108	45	[85]
	*Bacillus* sp.(B2)		50-37.06	74.1	[86]
	*Bacillus *sp.(B4)		50-36.57	73.14	
	*Bacillus *sp.(B9)		50-30.75	61.5	
	*Bacillus *sp.(B2)		100-42.15	42.15	
	*Bacillus *sp.(B4)		100-73.41	73.41	
	*Bacillus *sp.(B9)		100-60	60	
	*Bacillus *sp.(B4)		200-97.76	48.88	
	*Bacillus *sp.(B2)		200-81.5	40.75	
	*Bacillus *sp.(B9)		200-78.7	39.39	
	*Micrococcus *sp.		100	90	[87]
	*Acinetobacter *sp. B9(MTCC10506		16	78	[88]
			15	81	
	*Acinetobacter *sp. B9		7	67	[89]
			30	93.7	
			246	55.4	
	*Acinetobacter haemolyticus*		70	88	[90]
			100	75	
	*Acinetobacter *sp. (PCP3)		—	86	[91]
	*E.coli *( PCP1)		—	45	
	*Pseudomonas aeruginosa *(PCP2)		—	55	
	*Streptomyces* sp.		6.42	72	[85]
	Immobilized *B. subtilis* (B bead)		570-2	99.6	[92]
	Immobilized *P. aeruginosa* (P bead)		570-4	99.3	
	*Pseudomonas aeruginosa *(P)		570-2	99.6	[92]
	*Bacillus subtilis* (B)		570-2	99.6	
	*Stenotrophomonas *sp.		16.59	81.27	[93]

Bacteria	*Cellulosimicrobium* sp. (KX710177)	Pb	50	99.33	[94]
			100	96.98	
			200	84.62	
			300	62.28	
	*Methylobacterium organophilum*		—	18	[95]
	*Gemella* sp.		0.3	55.16±0.06	[96]
	*Micrococcus* sp.			36.55±0.01	
	*Bacillus firmus*		—	98.3	[97]
	*Pseudomonas* sp.		1	87.9	[98]
	*Staphylococcus* sp.		0.183	82.6	[85]
	*Streptomyces* sp.		0.286	32.5	[85]
	*B. iodinium*		100-1.8	87	[99]

Bacteria	*Desulfovibrio desulfuricans *(KCTC 5768) (immobilize on zeolite)	Cu	50	97.4	[76]
			100	98.2	
			200	78.7	
	*Staphylococcus *sp.		1.536	42	[85]
	*Streptomyces *sp.		1.129	18	[85]
	*Enterobacter cloacae*		100	20	[100]
	*Desulfovibrio desulfuricans *(immobilize on zeolite)		100	98.2	[76]
	*Bacillus firmus*		—	74.9	[97]
	*Flavobacterium *sp.		1.194	20.3	[85]
	*Methylobacterium organophilum*		—	21	[95]
	*Arthrobacter *strain D9		0.05	22	[101]
	*Enterobacter cloaceae*		100	65	[100]
	*Micrococcus* sp.		0.3	38.64±0.06	[96]
	*Gemella *sp.			50.99±0.01	
	*Micrococcus *sp.		0.3	38.64±0.06	[96]
	*Pseudomonas *sp.		1	41	[98]
	*Flavobacterium *sp.		0.161	25	[85]
	*A. faecalis *(GP06)		100-19.2	70	[99]
	*Pseudomonas aeruginosa *(CH07)		100-17.4	75	

Bacteria	*Desulfovibrio desulfuricans *(immobilize on zeolite)	Ni	50	90.3	[85]
			100	90.1	
			200	90.1	
	*Micrococcus *sp.		50	55	[87]
	*Pseudomonas *sp.		1	53	[98]
	*Acinetobacter *sp. B9		51	68.94	[89]

Bacteria	*Enterobacter cloacae*	Co	100	8	[100]

Bacteria	*Klebsiella pneumoniae*	Hg	100	28.65	[102]
	*Pseudomonas aeruginosa*		150	29.83	
	*Vibrio parahaemolyticus *(PG02)		5	90	[100]
			10	80	
	*Bacillus licheniformis*		0.1	73	[103]
	*Vibrio fluvialis*		0.25	60	[104]

Bacteria	*Bacillus firmus*	Zn	—	61.8	[97]
	*Pseudomonas *sp.		1	49.8	[98]

Consortium Organisms	*Acinetobacter *sp*. & Arthrobacter *sp.	Cr	16	78	[99]
	*P. aeruginosa & B. subtilis *(P+B)		570-2	99.6	[92]
	*S. cerevisiae & B. subtilis *(Y+B)		570-16	97.2	
	*S. cerevisiae & P. aeruginosa *(Y+P)		570-4	99.3	

Consortium Organisms	*B. licheniformis & C. parapsilosis*	Hg	0.1	85	[103]
	*C. parapsilosis & T. rostrata*			77	
	*B. licheniformis & T. rostrata*			73	
	*B. licheniformis, C. parapsilosis & T. rostrata*			88	

Fungi	*Aspergillus *sp.	Cr	100	92	[87]
	*Aspergillus versicolor*		50	99.89	[105]
	Immobilized* S. cerevisiae *(Y bead)		570-0	100	[92]
	*Gloeophyllum sepiarium*		—	94	[106]
	*Saccharomyces cerevisiae* (Y)		570-25	95	[92]
	*Aspergillus niger *(FIST1)		—	64.7	[91]
	*Mutant S. cerevisiae*		—	98.7	[56]
	*Sphaerotilus natans*		200	60	[107]
	*Saccharomyces cerevisiae*		—	99	[108]
	*Sphaerotilus natans*		200	82	[107]
	*Phanerochaete chrysosporium (immobilized on loofa sponge loofa sponge)*		100	98	[109]

Fungi	*Candida parapsilosis*	Hg	0.1	80	[103]

Fungi	*Aspergillus versicolor*	Cu	50	29.06	[105]
	*Aspergillus niger (* pretreated with Na2C03(0.2N))		20.82	41.7	[110]
	*Sphaerotilus natans*		200	58	[107]
	*A. lentulus*		100	99.7	[59]
	*Aspergillus niger*		—	50	[105]

Fungi	*Aspergillus versicolor*	Ni	50	30.05	[105]
	*Aspergillus *sp.		50	90	
	*Aspergillus niger *(pretreated with Na2C0390.2N))		—	40.5	[110]
	*Aspergillus niger*		0.38	98	[111]
	*Aspergillus niger*		—	58	[105]

Algae	*Spirogyra* sp.	Cr	5	98.23	[112]
	*Spirulina* sp.		5	98.3	[112]

Algae	*Chlorella vulgaris*	Pb	50 mg dm-3	99.4	[113]
	*Chlorella vulgaris*		51.79	99.4	[114]
	*Nostoc *sp.		1	99.6	[98]

Algae	*Chlorella vulgaris*	Cu	50 mg dm-3	97.7	[113]
	*Spirogyra *sp.		5	89.6	[112]
	*Spirulina* sp.		5	81.2	[112]

Algae	*Nostoc *sp.	Cd	1	95.4	[98]
	*Chlorella vulgaris*		50 mg dm^−3^	95.5	[113]
	*Nostoc *sp.	Zn	1	49.8	[98]

Algae	*Chlorella vulgaris*	Ni	0.6	41	[115]
	Nostoc sp.		1	88.23	[98]

Algae	*Nostoc* sp.	Fe	1	97.7	[98]

Microbial surfactants	P. aeruginosa ATCC9027(rhamnolipid)	Cd	22 *µ*g/mg	92	[116]

Protzoa	*Tetrahymena rostrata*	Hg	0.1	40	[103]

Microbial Fuel Cell (MFC)	Aerated microbial sediment fuel cells (A-SMFCs)	Cr	—	80.7	[117]
		Cu		72.72	
		Ni		80.37	
	Non aerated microbial sediment fuel cells (NA-SMFCs)	Cr		67.36	
		Cu		59.36	
		Ni		52.74	
